# World mental health day as an effective element of the public health system. 30 years of experience and perspectives

**DOI:** 10.1192/j.eurpsy.2025.1618

**Published:** 2025-08-26

**Authors:** V. F. Lebedeva, E. V. Gutkevich, O. A. Pavlova, S. V. Vladimirova, N. A. Bokhan, E. Mochalova

**Affiliations:** 1 Mental Health Research Institute, Tomsk National Research Medical Center, Russian Academy of Sciences; 2 Tomsk State University; 3 Siberian State University, Tomsk, Russian Federation

## Abstract

**Introduction:**

During Open Day at Mental Health Research Institute in Tomsk, assistance was provided to many citizens of Siberia.

**Objectives:**

to develop its algorithm.

**Methods:**

An analysis of experience in holding Mental Health Day.

**Results:**

Stage 1 – Preparatory: assessment of the prevalence of mental disorders; identification of the social challenges in the population that influence the growth of various forms of mental disorders; features of the functioning of general somatic health care services and psychiatric institutions, and their interaction.

Stage 2 – Information and methodological: conducting psychoeducational activities. The main topics are understanding mental health, the main risks, the first symptoms, ways to seek help. The anonymity, the free provision of services, the date, the form and method of the project are emphasized.

Stage 3 – Formation of the volume of services. A registration is made via telephone and electronic communications for those wishing to receive this help, indicating a specific and convenient time. This form of preliminary registration turned out to be logistically successful, aesthetically humane even under the conditions of sanitary and epidemiological restrictions of the COVID-19 pandemic.

Stage 4 – Implementation of Mental Health Day: on Saturday, in the week 1 of October. Filling in the express questionnaires. Formation of the main flows by specialists: 1) with affective disorders, 2) schizophrenia, 3) psychosomatic disorders, 4) post-traumatic stress disorders, 5) with addictions to alcohol, psychoactive substances, 6) persons of gerontological age. Methods of assistance: advisory, therapeutic (inpatient, outpatient), psychological support, provision of social services.

Stage 5 – Assessing the effectiveness of logistics and the results of Open Day based on the results of a consultation, analysis of questionnaires, verification of mental disorders, assessment of the main risk factors, identification of vulnerable groups of the population for the development of mental disorders.

Stage 6 – Achieving the goal of the World Mental Health Day project.

**Conclusions:**

An analysis of experience in holding Mental Health Day allowed us to develop its algorithm.

**Disclosure of Interest:**

None Declared

